# Supplementary material for: The causal relationship between the human gut microbiota and pyogenic arthritis: a Mendelian randomization study
Source: Front Cell Infect Microbiol. 2024 Nov 26;14:1452480. doi: 10.3389/fcimb.2024.1452480 (PMC11629706; doi:10.3389/fcimb.2024.1452480)
Supplement: Supplementary file 1 [file DataSheet1.zip › mendelian test/ebi-a-GCST90027787.csv_leave_one_out.pdf]

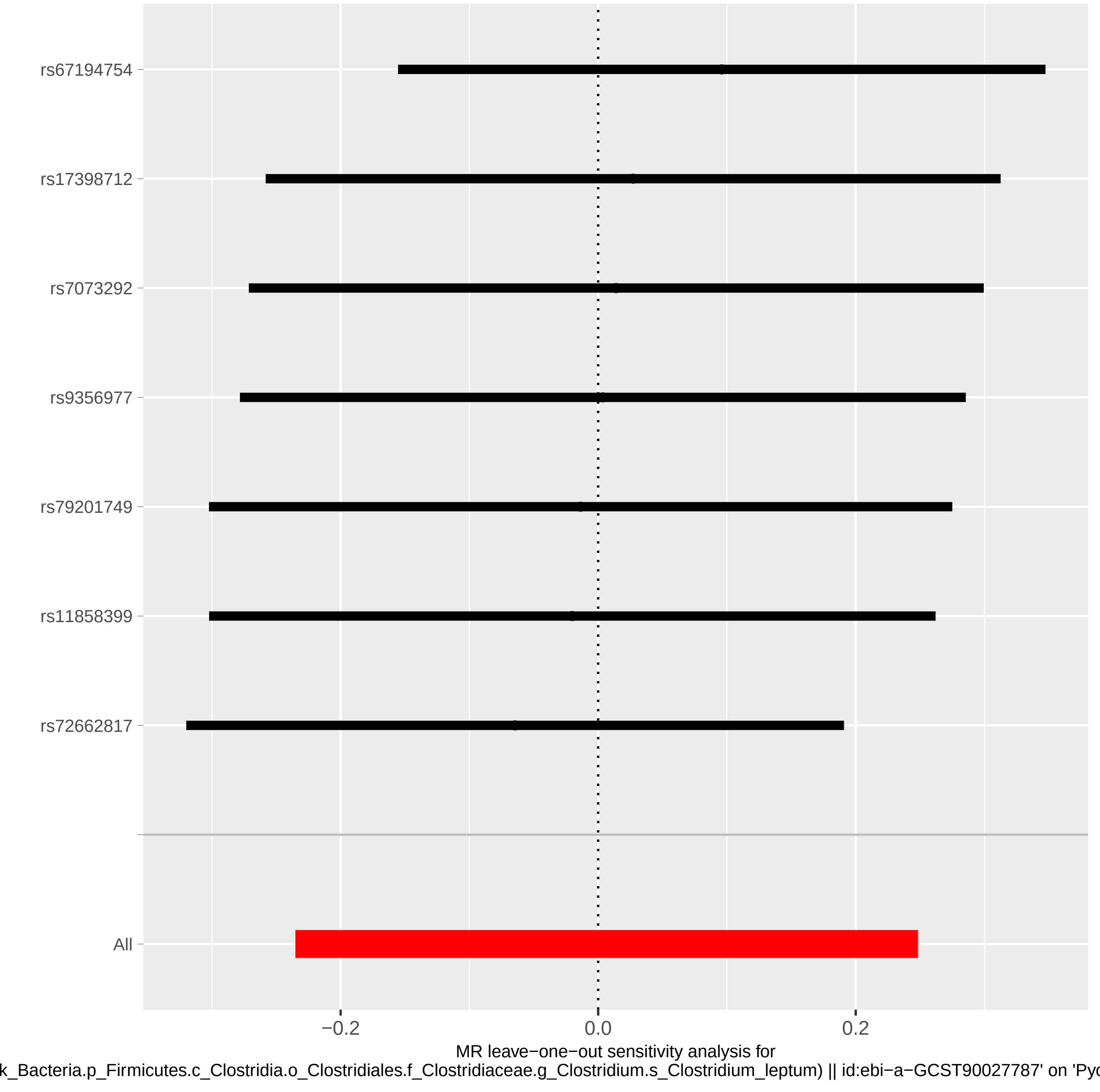

rs67194754
rs17398712
rs7073292
rs9356977
rs79201749
rs11858399
rs72662817
All
−0.2
0.0
0.2
MR leave−one−out sensitivity analysis for
k_Bacteria.p_Firmicutes.c_Clostridia.o_Clostridiales.f_Clostridiaceae.g_Clostridium.s_Clostridium_leptum) || id:ebi−a−GCST90027787' on 'Pyo
